# Understanding Error Culture in Veterinary Medicine: A Survey Among Veterinary Support Staff Across German-Speaking Countries

**DOI:** 10.3390/vetsci13030265

**Published:** 2026-03-13

**Authors:** Corinna M. Montag, Christin Kleinsorgen, Holger A. Volk, Claudia Busse

**Affiliations:** 1 Department of Small Animals Medicine and Surgery, University of Veterinary Medicine Hannover, 30559 Hannover, Germany; 2 Centre for Teaching, E-Learning-Services, University of Veterinary Medicine Hannover, 30559 Hannover, Germany

**Keywords:** error culture, veterinary support staff, error reporting, error prevention, error management

## Abstract

Errors are an unavoidable part of veterinary practice; however, little is known about how veterinary support staff perceive and deal with errors in their veterinary teams. This study examined perception of errors, contributing factors, and approaches to error management among veterinary support staff in German-speaking countries. A cross-sectional online survey was conducted, including 205 fully completed questionnaires. Reported errors were most often linked to tasks such as billing, team interaction, and handling and restraining animals. The most frequently reported contributing factors were time pressure, high workload, and communication problems. Most respondents reported that errors are openly spoken up about in everyday work (68%) and discussed within the team (76%). At the same time, perceptions of feeling safe to report an error varied: while 69% reported always or mostly feeling safe to speak up, 31% felt safe only sometimes, rarely or never. In addition, 16% of participants reported having deliberately not disclosed an error. According to 81% of respondents, no structured error reporting or management system was in place in their workplace. Overall, the findings show that error handling among veterinary support staff remains largely informal and individual, highlighting an important area for further research, improved reporting structures and training.

## Simple Summary

Veterinary practices aim to provide safe care for animals, yet errors can still occur in everyday work. Understanding how these errors happen and how teams deal with them is important for improving patient safety. However, little is known about how veterinary support staff experience and handle errors in practice. In this study, more than 200 veterinary support staff from German-speaking countries completed an online survey. The aim was to explore where errors are most likely to occur, which factors contribute to them, and how teams deal with them. Participants most often linked errors to administrative tasks such as billing, communication within the team, and handling animals. Time pressure, heavy workload, and communication problems were reported as the most common contributing factors, reflecting common human factors that influence work performance. While many respondents said that errors are discussed openly, most workplaces lacked structured systems for reporting and reviewing them. The findings suggest that better communication, supportive teamwork, and structured ways of learning from errors could help improve patient safety in veterinary practice.

## 1. Introduction

Errors are an inherent part of everyday professional life in healthcare, yet how they are dealt with has far-reaching consequences, not only for patients and animal owners, but also for healthcare professionals themselves. In human medicine, the examination of errors, their systemic nature, and the emotional experience associated with them has been subject of research for many years and plays an important role in patient safety research [1]. Within this context, the analysis of medical errors is regarded as a central component of patient safety efforts aimed at reducing preventable harm [2]. In comparison, the veterinary field has been less systematically examined with regard to patient safety and error prevention, despite the fact that the pressures, decision-making responsibilities, and ethical dilemmas are in many respects comparable with those of other health care sectors [3,4].

The veterinary workforce operates under conditions that differ fundamentally in several respects from those of other healthcare professions, which can significantly influence openness in error disclosure and the handling of medical errors [3]. Structurally, many veterinary practices are characterised by small teams and a high workload [5]. As described by Kinnison et al. (2014), work is typically organised around a close-knit veterinary team (hereafter “veterinary team”), consisting of veterinarians and veterinary support staff who work closely with them on a day-to-day basis [6]. Within these teams, veterinarians and veterinary support staff, such as veterinary nurses, patient care assistants, receptionists and administration staff, often take on several roles at the same time, including clinical care, diagnostics, communication with clients, and administrative tasks. This combination of multiple responsibilities and limited formal procedures, such as incident reporting systems, standardised checklists, or structured processes for error review and follow-up, can increase the risk of individual errors and make it harder to review and learn from them systematically [7]. In addition, work in veterinary teams often takes place within clear hierarchical structures [7]. Veterinary support staff are frequently directly dependent on practice owners or employed veterinarians, which may lead to a potential reluctance to speak up about critical incidents. Such organisational conditions are central to error culture, understood here as the shared norms and practices that shape how errors are perceived, communicated, and addressed within veterinary teams [8,9]. While systematic research specifically examining the role of hierarchy in veterinary error culture is still lacking, initial findings from veterinary studies [5,7], as well as evidence from human healthcare [9,10], indicate that authoritarian structures and low levels of psychological safety can hinder open communication about errors.

Veterinary teams face a range of challenges with communication in everyday practice [10,11]. Communication with animal owners is a central component of veterinary care and is carried out not only by veterinarians but also by veterinary support staff, often in emotionally demanding situations and under time pressure [11]. In many practice settings, this communication takes place without clearly defined structures or support from additional professional roles, such as practice management or quality management. At the same time, internal communication within veterinary teams has been described as predominantly informal, time-constrained, and only limitedly documented [12]. This highlights the need to systematically examine communication practices and error culture within veterinary teams. Understanding how errors are discussed, which communicative and organisational structures and cultures are perceived as supportive or hindering, and how team members experience psychological safety are important areas to study. In this context, a targeted examination of these conditions appears to be a key prerequisite for safeguarding both the wellbeing of veterinary support staff and the long-term quality of veterinary care. With veterinary teams, the concept of the “second victim” is relevant for both veterinarians and veterinary support staff, as it describes the often overlooked emotional suffering experienced following errors or critical incidents [13,14]. While patient safety and the prevention of harm to the primary victim, the patient, remain central, the term “second victim” is used here descriptively to refer to the psychological impact experienced by professionals involved in adverse events, rather than to imply equivalence of harm. While this phenomenon has been increasingly discussed in human healthcare and, to some extent, in veterinary medicine, existing research has predominantly focused on veterinarians. In contrast, empirical evidence on the emotional impact of errors on veterinary support staff remains scarce, despite indications of considerable psychological strain. Preliminary findings from a wellbeing survey suggest that veterinary nursing staff, in particular, experience pronounced stress symptoms and reduced wellbeing [15]. In addition, research on occupational stress in veterinary nurses demonstrates that working conditions and organisational factors are key contributors to emotional strain and burnout [16].

The purpose of this study is to examine error management and workplace conditions among veterinary support staff working in veterinary teams, with a particular focus on identifying frequently perceived error areas, analysing structural and work-related factors that contribute to their occurrence, and exploring how errors are addressed at both the individual and team level.

## 2. Materials and Methods

### 2.1. Study Design and Questionnaire Development

The questionnaire used in the present study was conceptually adapted from a previously published survey on error culture of veterinary surgeons [17]. Building on the conceptual framework of close-knit veterinary teams (hereafter “veterinary team”), as described by Kinnison et al. (2014), the questionnaire was adapted for use with veterinary support staff working in clinical practice, who form an integral part of day-to-day veterinary team structures [6]. While the overall structure and key thematic areas of the questionnaire were retained, selected items were reworded, supplemented, and reorganised to more fully capture aspects of workplace atmosphere and perceived psychological safety when raising errors from the perspective of veterinary support staff. In line with the composition of veterinary teams, the survey addressed individuals working in a range of practice-based support roles within veterinary practices, including veterinary nurses, animal care assistants, personnel in management roles and other support roles involved in daily clinical or organisational workflows. In Germany, the profession of veterinary nurse is nationally recognised and regulated, requiring completion of a three-year vocational training programme [18]. However, unlike veterinarians, they are not subject to an independent professional regulatory body or disciplinary system. In everyday practice, they typically work as employed staff and carry out delegated tasks under the professional responsibility of a supervising veterinarian.

Throughout the questionnaire, the term “error” was used instead of “mistake”, as it represents the established terminology in healthcare and patient safety research and more appropriately reflects clinical, procedural, and systemic dimensions of veterinary practice [19]. In line with patient safety literature, the term encompasses adverse events resulting in harm, near misses, and no-harm incidents, that is, deviations from intended processes with potential or actual relevance to patient safety [5]. In the survey, the term error was not explicitly defined in order to capture the individual experiences of participants and to avoid influencing their understanding of the concept.

### 2.2. Content Structure and Design

The final questionnaire comprised a total of 22 questions and was structured into four main thematic sections, from general background information to more specific aspects of error occurrence and workplace culture.

The first section collected general professional background information. These questions served to characterize the respondents regarding their vocational training status, years of professional experience, type of workplace, and professional role. The items were presented in a single-choice format to allow clear categorization of participants.

The second section focused on the occurrence and handling of errors in daily work. This section explored the self-perceived frequency of errors, activities in which errors were considered most likely to occur, contributing factors, and the role of workload, communication, and the working environment. In addition, respondents were asked how errors are handled individually and within the team, whether structured error reporting or management systems exist in their workplace (not further specified in the questionnaire), and how well their education or training prepared them to deal with errors. An optional free-text question allowed participants to provide suggestions for error prevention.

The third section addressed aspects of workplace climate and practice culture. Participants were asked to assess the general working atmosphere, perceived work-related stress, and their own overall job satisfaction. An optional free-text question invited respondents to share suggestions for improving the working atmosphere, allowing for qualitative insights beyond predefined response categories.

The fourth section collected basic demographic information, including gender and year of birth. This information was used to further describe the study population.

### 2.3. Study Population and Inclusion Criteria

Included were members of the defined group of veterinary support staff who were actively working in clinical practice at the time of data collection. Recruitment took place in the German-speaking region. Prior to accessing the questionnaire, participants were provided with written information about the study aims, voluntary participation, and data protection. Proceeding with the online survey was considered to indicate informed consent. The complete questionnaire is provided in Supplementary Material S1: Survey Questionnaire. Participants who did not meet the inclusion criteria, such as veterinarians, were excluded from the analysis. After completion of data collection, the datasets were cleaned and checked for accuracy, plausibility, and completeness. Incomplete responses were excluded from analysis.

### 2.4. Data Collection

The anonymous online questionnaire was implemented using LimeSurvey (Community Edition Version 6.13.2+250506, LimeSurvey GmbH, Hamburg, Germany) and distributed in July 2024. Recruitment was carried out via the social media accounts of the University of Veterinary Medicine Hannover, the social media initiative “fehlerkultur_tiermedizin”, the platform “tfa.portal”, as well as via email distribution to clinical staff across the German-speaking region.

### 2.5. Data Analysis

The dataset was exported from LimeSurvey and analysed using Microsoft Excel for Mac 2024 (Microsoft Corp., Redmond, WA, USA). Descriptive statistical methods were used to summarise the results and describe response patterns; no inferential statistical testing was conducted. All percentages were rounded to whole numbers (zero decimal places) in accordance with the survey output format. One ranking question assessed which activities were perceived as most frequently associated with errors. Participants ranked between three and eleven predefined activities using a drag-and-drop function. Participants were not required to rank all items. Responses were assigned points based on their rank position, with higher-ranked items receiving higher point values. The results were analysed based on the total weighted scores, allowing identification of the activities that were prioritised by respondents as being associated with errors. Free-text responses addressing suggestions for error prevention were analysed using reflexive thematic analysis following the approach described by Braun and Clarke (2014) [20]. Themes were developed to capture shared meanings across participants’ responses rather than merely sorting statements into categories. The analysis involved familiarising with the data, coding and developing and refining the themes and was conducted manually. The research team consisted of professionals with backgrounds in veterinary medicine and veterinary education, and reflexive consideration was given to how this positioning may have informed the analytic process. In contrast, free-text responses concerning improvement of the working atmosphere were analysed descriptively, as this question served a supplementary purpose and was not intended for in-depth thematic development within the central research focus on error perception and error culture. All free-text responses were translated into English for publication. Group comparisons were conducted descriptively and exploratively; no inferential statistical testing was performed.

## 3. Results

### 3.1. Participants’ General and Demographic Data

Among all participants (*N* = 205), 97% identified as female, while 1% identified as male and 2% did not specify their gender. Participants’ ages ranged from 17 to 60 years, with a median age of 29 years and the majority of respondents were within the category of 21–30 years (49%). Most respondents reported having completed a formal vocational training (84%). A smaller proportion was still undergoing vocational training (11%), while a minority reported working in a veterinary setting without having completed any formal vocational qualification for veterinary support roles (4%).

In terms of professional role, most participants worked as veterinary nurses (89%). Smaller groups included patient care assistant (2%), management or administrative staff (4%), and other roles (5%). Most respondents (40%) had been working in the veterinary field for less than five years, and the majority (70%) reported working primarily in a private veterinary practice. Further details can be found in Table 1.

### 3.2. Occurrence and Handling of Errors

When asked how frequently errors occur in their daily work, 57% of respondents (*N* = 205) reported that this happens rarely, 41% occasionally, 2% often and none reported that errors never occur. The responses regarding the ranking of specific tasks associated with error occurrence reflect subjective perceptions rather than objective incidence rates.

**Figure 1:**
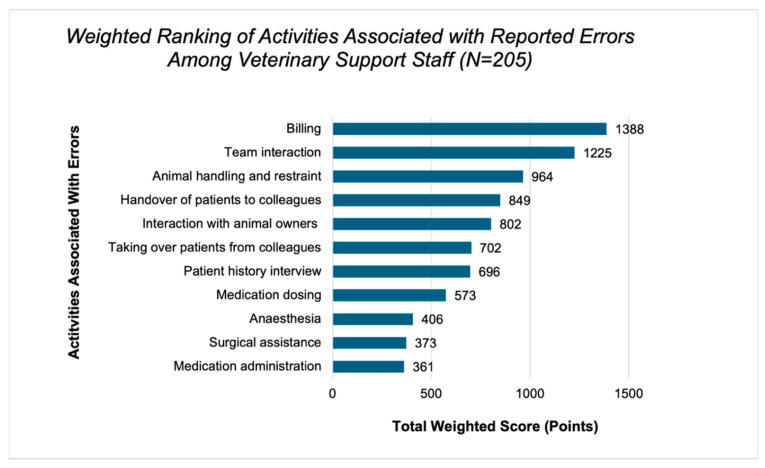
Weighted ranking of activities associated with self-reported errors among veterinary support staff who reported at least one error (*N* = 205). Respondents could select and rank between three and eleven activities. Values represent total weighted scores reflecting the relative prioritisation of activities across respondents.

According to the ranking, the three tasks most frequently associated with errors were billing, team interaction and animal handling/restraint (Figure 1).

**Figure 2:**
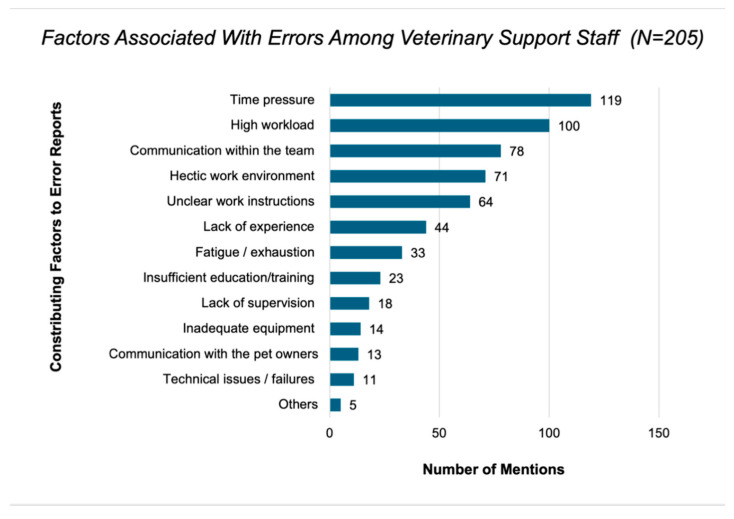
Perceived contributing factors to self-reported errors among veterinary support staff (*N* = 205, 100% of all participants). Participants could select up to three factors. Bars indicate the number of respondents mentioning each factor.

Further, the following three factors most commonly contributing to the occurrence of errors were time pressure, high workload and team communication (Figure 2).

Among all participants (*N* = 205), different ways of dealing with errors were reported at the individual and team level. At the individual level, most participants reported that they address errors openly (68%, *n* = 140). Many also stated that they try to correct errors themselves (55%, *n* = 113) or reflect on them afterwards (45%, *n* = 92). In contrast, 16% reported having previously concealed an error (*n* = 33). At the team level, errors were most frequently described as being openly addressed and analysed within the team (76%, *n* = 156). In comparison, 14% of respondents stated that errors are covered up within their team (*n* = 28), while 22% reported that errors are followed up in conversations with supervisors (*n* = 46).

**Figure 3:**
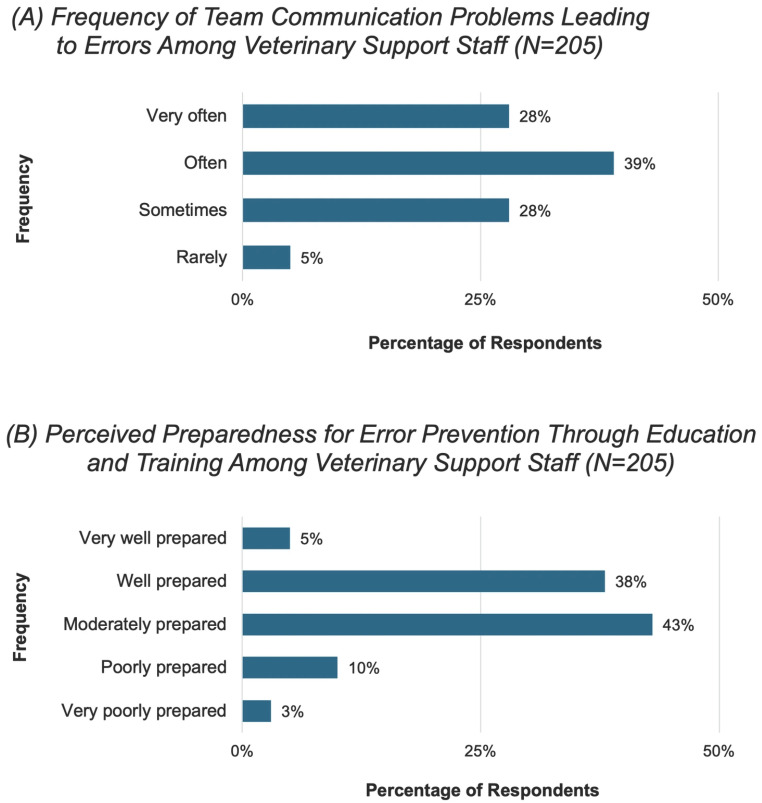
Frequency of self-reported team communication problems leading to errors among veterinary support staff (**A**) and their self-reported perceived preparedness for error prevention through education and training (**B**) among veterinary support staff (*N* = 205, 100% of all participants).

When asked how safe participants felt speaking up about errors within their team, 69% reported that they *always* or *mostly* felt safe (*n* = 141). The remaining participants reported feeling *sometimes* (16%, *n* = 33), *rarely* (13%, *n* = 27), or *never* (2%, *n* = 4) safe to speak up about errors. Regarding how often communication problems within the team lead to errors, most participants reported that communication problems lead to errors *very* or *rather often* (67%, *n* = 137) (Figure 3A).

When asked about the presence of a structured error reporting and management system, most participants reported that no such system exists in their workplace (81%, *n* = 167) or that they did not know whether such a system exists (13%, *n* = 27). Only a small number confirmed the presence of such a system (5%, *n* = 11).

In response to the question of how much workload contributes to the occurrence of errors, most participants reported that workload plays a large (49%, *n* = 101) or very large role (36%, *n* = 74).

Regarding the working environment, including physical space and technical equipment, 49% of participants reported that it *partly* contributes to the occurrence of errors (*n* = 101), while 11% reported a *strong* contribution (*n* = 22). Others rated its influence as *neutral* (25%, *n* = 51) or *small* (13%, *n* = 26) or no influence at all (2%, *n* = 5).

When asked how well their education and training prepared them to prevent errors, 43% (*n* = 89) rated their preparation as *moderate* and 38% (*n* = 77) as *well prepared* (Figure 3B).

### 3.3. Qualitative Analysis of Free-Text Responses

A thematic analysis of free-text responses from 77 participants (38%) to the question “What do you think would be good suggestions for preventing errors?” identified six interrelated themes concerning error prevention in veterinary practice. The themes were developed interpretatively to capture shared patterns of meaning across responses and are presented in a data-near format to reflect participants’ perspectives transparently. Below, the themes are presented with illustrative examples drawn directly from participants’ responses:

A central pattern across responses concerned communication, which was also the most frequently identified theme. Participants emphasised that open, clear, and respectful communication within the team plays a central role in preventing errors. This included communication on an equal footing, clarity of instructions, and explicit clarification of responsibilities. Participants highlighted the importance of written and verbal communication, particularly from supervising veterinarians, as well as a non-blaming and calm approach when discussing errors. Statements such as “Better team communication without blame but focused solely on solutions” (ID#59) or “Open communication within the team; clear instructions (possibly in written form)” (ID#18) illustrate this emphasis.

Closely connected to communication, participants highlighted the importance of structures and standardised procedures. Participants emphasised the importance of clear, written instructions, checklists, and predefined workflows to support consistent and safe practice. Standardisation was described as particularly helpful in complex or repetitive tasks and in clarifying responsibilities. For example, respondents referred to “Defined protocols, regular reminders” (ID#46), “Standardised and not constantly changing work processes” (ID#48) and “Optimising the working environment (office separated from reception; communication by management/introduction of structures for dealing with errors)” (ID#41) as practical means of reducing error risk.

Another recurring dimension concerned workload and time pressure as contextual conditions influencing safe practice. Participants suggested that excessive workload and insufficient recovery time increase the likelihood of errors.

Suggestions such as creating “A calm working environment and scheduling consultations in a way that allows sufficient time for each patient without creating stress” (ID#33), as well as calls for “More recovery time/breaks” (ID#75) and a “Smaller workload, reduced time pressure” (ID#78), reflect the perception that organisational demands influence attentiveness and performance in everyday practice.

Training and reliable supervision, especially during induction phases, also emerged as a meaningful pattern. Longer onboarding periods, patient and well-trained supervisors, and protected learning time were described as important in relation to error prevention, particularly among less experienced staff. For instance, respondents suggested “Good onboarding with a patient person who does not take things personally and explains calmly when something is done incorrectly” (ID#65) and referred to the need for “Preparation for conversations with the supervisor without fear of consequences” (ID#52) and proposed that “veterinarians were trained in staff management before becoming self-employed” (ID#7).

Participants also frequently referred to the importance of an open and learning-oriented error culture. Errors were described not as individual failures but as opportunities for collective learning and improvement. Open discussion of both actual and potential errors, team-based analyses, and structured reflection formats were described as important elements in relation to preventing future errors. This perspective was reflected in statements such as “Error analyses within the team, further training especially for younger colleagues, improvement of communication among each other; unfortunately, among many colleagues the trend has for years been more towards ‘concealing errors, not making oneself vulnerable, perceiving constructive criticism as an attack …’ instead of wanting to work on oneself and accept help to avoid errors” (ID#17), as well as references to “Error analysis, M&M rounds, time for communication during handovers, handover protocols” (ID#73). Participants further emphasised “An open way of working together in which no one feels attacked” (ID#45) and suggested that “An open approach to dealing with problems and fixed structures in workflows would prevent errors” (ID#10).

Finally, teamwork was described as a protective factor against errors, particularly when team members are aware of each other’s strengths and limitations and support one another across hierarchical levels. Respectful interaction, mutual support, and inclusion of trainees were described as key components of effective teamwork. These views were reflected in statements such as “Joint resolution of the error/the problem resulting from it. […] Working as a team so that there is no repetition. Clear instructions, possibly documented in writing (for non-routine matters). […]” (ID#54) and “Good teamwork, respectful interaction across all levels, good onboarding, communication, helping each other, sharing experiences” (ID#58).

Taken together, the qualitative responses suggest that participants conceptualise error prevention as emerging from the interaction between relational dynamics (communication, teamwork, error culture) and structural conditions (workload, procedures, training). While presented in a data-near manner, these patterns reflect interpretatively developed themes capturing shared meanings across responses.

### 3.4. Workplace Atmosphere and Practice Culture

All participants (*N* = 205) were asked to describe the current atmosphere in their workplace. Overall, 58% of all participants rated the atmosphere as very or rather positive (*n* = 119), while 23% reported a rather or very negative atmosphere (*n* = 47). In contrast, 19% described the atmosphere as neutral (*n* = 39).

Regarding perceived work-related burden, 34% of participants reported *high or very high* burden (*n* = 69). A further 52% reported *moderate* or *low burden* (*n* = 126), while only 5% reported no burden at all in their workplace (*n* = 10).

With regard to current job satisfaction, 48% of participants reported being satisfied or *very satisfied* with their current professional situation (*n* = 98). In contrast, 27% reported being *dissatisfied* or *very dissatisfied* (*n* = 55), while 25% rated their job satisfaction as *neutral* (*n* = 52).

Free-text responses were provided by 74 participants (36%) and were analysed descriptively to summarise frequently mentioned factors that participants perceived as contributing to an improved working atmosphere, as the focus was on general workplace conditions rather than on analytically developing themes related to error culture. Participants most often referred to communication and interpersonal interaction, particularly with supervisors. Participants mentioned honest and appreciative communication, as well as more team-building activities and regular discussions. In addition, workload and staffing levels were frequently raised, with participants expressing a need for more staff, regulated working hours, reliable breaks, and higher pay.

## 4. Discussion

The aim of this study was to examine how veterinary support staff in close-knit veterinary teams perceive errors in their daily work, how they deal with them, and which structural and work-related factors are associated with their occurrence, within the broader context of patient safety in veterinary medicine. Overall, respondents perceived errors as mostly rare or occasional and mainly linked them to everyday organisational and communication processes in veterinary practice. Time pressure, high workload and communication problems were identified as the most important influencing factors. Although errors are often discussed openly within the team, the perceived level of psychological safety varies, and some participants reported both actively withholding errors and the absence of structured error management systems. Overall, the results suggest that the handling of errors in daily practice is largely informal and strongly dependent on the individual practice.

### 4.1. Perception of Errors

In studies from human medicine and nursing, the occurrence of errors is often reported to be higher. In one study, more than 30% of nurses reported having made at least one error within a month [21]. The rare-to-occasional perception reported in the present study suggests that subjective assessments do not necessarily reflect the actual prevalence of errors. Instead, they reflect individual perceptions that are influenced by the existing error culture and by how errors are defined [22]. Errors were deliberately not defined in the questionnaire in order to avoid influencing participants. However, this open approach may also have influenced how frequently errors were perceived. Therefore, the focus of this study was on capturing subjective perceptions of errors in everyday work. In the literature, errors are not described as exceptions but as an inherent part of professional practice [1,6]. The free-text responses also suggest that errors are seen as part of everyday work and are actively reflected upon. Some participants suggested structured formats for jointly analysing errors, such as “Error analysis, M&M rounds … handover protocols” (ID#73). At the same time, it was noted that among colleagues there is sometimes a tendency to “conceal” errors and not make oneself “vulnerable” (ID#17). These statements show that errors are present in everyday work, but how they are handled is strongly shaped by the existing error culture.

### 4.2. Tasks and Contributing Factors Associated with Errors

Certain activities were perceived by participants as particularly prone to error. These include billing, team interaction, and handling and restraining animals. These activities combine clinical, organisational and communication demands and are therefore more complex. Client-facing administrative processes such as billing have been described as particularly vulnerable to errors where documentation, financial information, and interaction with animal owners coincide [12,23]. In safety research, interactions within the team are also described as particularly prone to error [24]. Handling animals is also a known safety challenge, as incidents such as falls, escapes or insufficient restraint are described as relevant events in studies on veterinary patient safety [25,26]. At the same time, occupational health studies show that animal bites and injuries are among the most common workplace accidents in veterinary medicine [27].

Time pressure and high workload were perceived as the main factors influencing the occurrence of errors. These factors are also well known in safety research as important conditions for systemic error processes [28]. Communication problems within the team were also reported by more than half of the participants as a relevant factor. In human medicine, communication in general is considered an important requirement for safe working processes [12]. Around half of the participants also considered physical working conditions and technical equipment as influencing factors. These findings can be understood using the “Systems Engineering Initiative for Patient Safety model” (SEIPS model), which describes the physical environment, available technologies and organisational conditions as interacting parts of a work system and assumes that errors are not mainly caused by individual mistakes [29]. In the free-text responses, participants suggested measures such as “clear instructions (possibly in written form)” (ID#18) and standardised processes (“Defined protocols, regular reminders”, ID#46; “Standardised … work processes”, ID#48). Improvements to the working environment were also mentioned (“Optimising the working environment … introduction of structures”, ID#41). In addition, time pressure and recovery time were directly addressed (“Smaller workload, reduced time pressure”, ID#78; “More recovery time/breaks”, ID#75; “scheduling … sufficient time … without creating stress”, ID#33).

### 4.3. Error Reporting and Handling of Errors

The results suggest that errors are already sometimes discussed openly by veterinary support staff, but that the way they are handled is still largely informal. More than half of the participants reported correcting errors themselves, while structured error reporting systems were not present in the working environments of more than 80% of respondents. Similar patterns have also been described in other areas of healthcare, where the lack of organisational structures can make learning at a system level more difficult [19,30,31]. In line with this, the free-text responses repeatedly suggested formal but non-punitive team formats (e.g., “Error analysis, M&M rounds …”, ID#73) and emphasised the importance of solution-focused communication about errors without blame (“Better team communication without blame, but focused solely on solutions”, ID#59).

### 4.4. Psychological Safety

Although more than 60% of respondents stated that they feel safe addressing errors, more than a quarter reported doing so only occasionally or not at all. At the same time, nearly half of the participants stated that they reflect on errors only privately, and around one in six participants reported actively concealing errors. This coexistence of open communication and selective withholding suggests that speaking up about errors may still depend strongly on the situation and organisational conditions and may indicate unstable psychological safety. Studies from human medicine show that hierarchical structures, fear of negative consequences and limited feedback structures can hinder open communication about errors [9,32,33]. In the free-text responses, a tendency to conceal errors or avoid vulnerability is described (“concealing errors, not making oneself vulnerable …”, ID#17), while at the same time, participants called for the possibility of speaking to supervisors without fear of consequences (“Preparation for conversations with the supervisor without fear of consequences”, ID#52). These findings highlight the importance of leadership and psychological safety for a learning-oriented error culture.

### 4.5. Working Atmosphere

Questions about working atmosphere and perceived workload among veterinary support staff were included to provide initial insights into the organisational structure of everyday practice. While many respondents described a positive working atmosphere, many also reported a high workload. Research from human medicine shows that working conditions and team climate are closely linked to staff well-being and to communication processes within the team [33,34]. The qualitative responses also highlighted the importance of respectful teamwork, supportive leadership and suitable working conditions for safe working, for example, through calls for “smaller workload, reduced time pressure” (ID#78), adequate staffing levels and “good onboarding … explaining calmly when something is done incorrectly” (ID#65). Overall, the results show that the error culture in veterinary practice, particularly from the perspective of veterinary support staff, is closely linked to organisational and interpersonal conditions.

### 4.6. Practical Recommendations

Based on the findings of the survey, the following recommendations can be summarised.

Error management should be established regardless of how frequently errors are subjectively perceived. Even rarely noticed errors and small deviations should be systematically recorded and reviewed in order to support learning and prevention. A shared understanding of what is considered an error could help with this, for example, by identifying and reflecting on such events at an early stage. This recommendation is based on the discrepancy observed in this sample between the subjective perception of errors and the higher error frequencies reported in patient safety research, as well as on the important role of structured event reporting for learning processes [1,35,36,37].

Based on the findings about error-prone tasks, it seems useful to pay particular attention in daily practice to billing, team interaction, and the handling and restraining of animals. Considering the high frequency of animal-related workplace accidents reported in previous studies, strengthening safety awareness and providing targeted training in these areas may also help to reduce injuries. Regular internal team discussions, such as morbidity-and-mortality conferences or short case reflections on typical problem situations, including possible errors recognised by staff, could also help identify recurring sources of error and develop solutions together. Such formats allow teams to learn from everyday experiences and integrate error prevention more strongly into daily work processes and other activities in which errors may occur.

With regard to contributing factors, the results of this study are largely consistent with findings from patient safety research showing that working conditions and work organisation can influence the occurrence of errors in everyday work [28,29,38]. It therefore seems useful to regularly review and adjust these organisational conditions in veterinary practices. This includes work processes, task distribution, available time resources and the design of the working environment. Practical measures could include regular reviews of workload, staffing levels and communication structures, especially during periods of increased time pressure.

With regard to reporting and dealing with errors, informal conversations about errors and individual corrections could be complemented by formal, non-punitive error reporting and error management structures. Such structures could help move from an individual approach to a shared learning process at the team or organisational level, without replacing open communication within closely working veterinary teams [19,30,31]. Established models from human medicine may serve as examples, such as the CIRSmedical programme of the German Medical Association, which has allowed anonymous reporting and structured case analysis since 2008 [39]. In veterinary medicine, existing approaches such as internal reporting systems in larger practice networks or quality-related programmes should be systematically reviewed, adapted and further developed [40]. In addition, structured and regularly scheduled formats for joint error analysis are recommended, such as the previously mentioned case discussions or morbidity-and-mortality conferences used in human medicine. These formats can provide a clear and non-punitive framework for analysing errors and adverse events and for taking action based on the insights gained [41]. Current veterinary research also highlights the importance of structured morbidity-and-mortality meetings for categorising events and strengthening patient safety culture in veterinary practices [2].

Education and training opportunities on error prevention and team communication should be actively promoted to strengthen patient safety. As preparation for dealing with errors was sometimes perceived as only moderate, and there are indications that psychological safety within teams may not always be stable, it seems useful to strengthen these competencies further. Training on error prevention, team communication or time management could therefore be more strongly integrated into both vocational education and continuing professional development [42]. Such measures are likely to be most effective when embedded in a supportive organisational climate, as this can promote psychological safety and open learning within teams [9]. In veterinary practices, leadership also plays an important role, as leaders can strongly influence whether staff feel safe speaking about problems or errors through their communication style and their approach to mistakes [32,33]. This includes both veterinary support staff and veterinarians, who play a key role in team leadership and work organisation.

Recommendations regarding working atmosphere and job satisfaction should be understood in the context of the existing literature and the previous sections and should complement the recommendations made there. It may be useful to regularly reflect on the working atmosphere, workload and job satisfaction and consider them as part of existing quality and safety measures [9,33,38]. As contextual factors, they may support the use and long-term stability of the previously recommended non-punitive structures for error communication and error management, without themselves being seen as independent tools for error prevention.

## 5. Limitations of This Study

Several limitations should be considered when interpreting the findings of this study. First, the data are based on self-reported assessments, which may be influenced by subjective perceptions and recall bias. In addition, participation in the survey was voluntary, introducing the possibility of self-selection bias. Individuals with a particular interest in patient safety or error-related topics may therefore be overrepresented. As a result, the findings may not fully reflect the views of all veterinary support staff, particularly regarding the prevalence of specific experiences or attitudes. However, such selective participation primarily affects representativeness, while patterns and associations observed within the participating group can still be informative [43]. The survey was primarily distributed via social media and professional veterinary networks, which may have limited the diversity of the sample and restricted participation to individuals who are digitally engaged or well-connected within professional communities.

A further limitation relates to the demographic composition of the sample. The large majority of respondents identified as female, which likely reflects the gender distribution within veterinary support professions in German-speaking countries [44]. At the same time, women are generally more likely to participate in questionnaire-based research, particularly on reflective topics such as error reporting, which may further contribute to their overrepresentation and should be considered when interpreting the findings [43].

Moreover, the study focuses exclusively on veterinary support staff in German-speaking countries. Cultural, organisational, and regulatory differences may therefore limit the transferability of the findings to other national or healthcare contexts.

Finally, the original questionnaire was translated from German into English, and despite careful translation, minor shifts in nuance or meaning cannot be completely ruled out. Moreover, responses may have been subject to question order effects, including primacy and recency effects, which are known to influence questionnaire-based data and cannot be ruled out.

## 6. Future Research

The findings of the present study give rise to several specific directions for future research. One key area concerns the emotional and psychological consequences for veterinary professionals following errors. Given that errors are often managed on an individual level, further research is needed to explore how errors are emotionally processed and which forms of support or individual coping strategies are effective in everyday practice. Another area concerns the differentiation between different types of errors. In the present study, the broader term “error” was used in line with patient safety research and was intentionally not further defined in the questionnaire in order to capture participants’ own experiences and avoid influencing their interpretation. Future studies could therefore distinguish more explicitly between adverse events, near misses, and no-harm incidents in order to gain a more detailed understanding of how different types of events are recognised, reported, and managed in veterinary practice. A further area relates to the development and evaluation of structured error reporting and learning systems in veterinary medicine. While the results indicate a widespread lack of formalised structures, future studies should examine which reporting formats—anonymous, open, or combined—are feasible in veterinary settings and to what extent such approaches are acceptable, feasible, and effective in supporting organisational learning. In addition, further research is required in the field of education and training. Although the present study highlights the relevance of perceived preparedness, there is a need for systematic analyses of how topics such as error management, communication, and working under pressure are embedded in veterinary curricula, as well as how well individuals currently enrolled in formal vocational training and early-career professionals feel prepared for clinical practice. Together, these lines of research may contribute to evaluating and further developing an evidence-based and practice-oriented error culture in veterinary medicine.

## 7. Conclusions

This study provides insight into how veterinary support staff in close-knit teams in German-speaking countries experience and handle errors in everyday practice. The findings show that errors are generally perceived as occurring rarely or occasionally and are mainly associated with organisational tasks such as billing, teamwork, and the handling and restraint of animals. Time pressure, workload, and communication problems were reported by participants as important contributing factors. Error handling in many teams is characterised by a willingness to speak openly about errors; however, this openness is not consistently supported by formal structures and therefore remains largely informal and dependent on individual responsibility or local team practices. Taken together with existing patient safety research, these findings indicate that the current error culture among veterinary support staff contains a valuable degree of openness that could be further developed and supported by appropriate organisational structures. Building on this existing openness, structured and non-punitive approaches to error reporting and joint error review may help support learning from everyday errors, particularly when errors are addressed and reflected on a regular and repeated basis. The findings also highlight the importance of preparation and training, as well as supportive leadership behaviour and psychologically safe working environments, in enabling open communication about errors. Overall, the results suggest that combining existing openness with supportive organisational conditions and opportunities for reflection may help to strengthen how errors are addressed in everyday veterinary practice.

## Figures and Tables

**Table 1:** Distribution of respondents (*N* = 205, 100% of all participants) according to age, vocational training status, years of professional experience, professional role within the close-knit veterinary team, and type of workplace.

**Age Distribution **		
**Answers**	**Percentage**	**Responses**
17–20 Years	8%	*n* = 16
21–30 Years	49%	*n* = 100
31–40 Years	23%	*n* = 48
41–50 Years	13%	*n* = 27
>50 Years	7%	*n* = 14
**Vocational Training Status**		
**Answers**	**Percentage**	**Responses**
Completed Formal Vocational Training	84%	*n* = 173
Currently Enrolled in Formal Vocational Training	11%	*n* = 23
No Formal Vocational Qualification	4%	*n* = 9
**Years of Professional Experience**		
**Answers**	**Percentage**	**Responses**
<5 Years	40%	*n* = 81
6–10 Years	29%	*n* = 59
11–15 Years	11%	*n* = 23
>15 Years	20%	*n* = 42
**Professional Role Within the Close-knit Veterinary Team**		
**Answers**	**Percentage**	**Responses**
Veterinary Nurses	89%	*n* = 182
Practice Management/Administration	4%	*n* = 8
Patient Care Assistant	2%	*n* = 4
Other	5%	*n* = 11
**Type of Workplace**		
**Answers**	**Percentage**	**Responses**
Practice (primary care facilities without 24 h emergency services)	70%	*n* = 144
Clinic (facilities providing 24 h emergency care)	20%	*n* = 40
Small Animal Centre (larger outpatient facility without 24 h emergency care)	7%	*n* = 15
Academic Institution (e.g., veterinary schools or university hospitals)	<1%	*n* = 2
Other	2%	*n* = 4

## Data Availability

The data presented in this study are available on request from the corresponding author. Data are available upon request and are not publicly available due to data protection policy.

## Supplementary Materials

The following supporting information can be downloaded at: https://www.mdpi.com/article/10.3390/vetsci13030265/s1, Table S1: Survey Questionnaire.

## Abbreviations

The following abbreviations are used in this manuscript:

CIRS	Critical Incident Reporting System
M&M	Morbidity and Mortality
N	Number of participants/respondents
SEIPS	Systems Engineering Initiative for Patient Safety
