# Supplementary material for: Understanding Error Culture in Veterinary Medicine: A Survey Among Veterinary Support Staff Across German-Speaking Countries
Source: Vet Sci. 2026 Mar 13;13(3):265. doi: 10.3390/vetsci13030265 (PMC13029886; doi:10.3390/vetsci13030265)
Supplement: Supplementary file 1 [file vetsci-13-00265-s001.zip › vetsci-4183978-supplementary.pdf]

Question Group	*Survey Question and Answer*	*Question Type*
Professional Background (N=205, 100%)		
	Have you already completed your training?<br>• Yes<br>• No, I am still in training<br>• No, I have not completed any formal training	Single choice
	How long have you been working in veterinary medicine?<br>• < 5 years<br>• 6–10 years<br>• 11–15 years<br>• > 15 years<br>• I am no longer professionally active	Single choice
	What type of facility do you mainly work in?<br>• Practice<br>• Small animal centre<br>• Clinic<br>• Academic institution<br>• Research institution<br>• Zoo / wildlife park<br>• Other	Single choice
	Which professional group do you belong to?<br>• Veterinary nurse<br>• Animal care assistant<br>• Management<br>• Other	Single choice
Dealing with Errors (N=205, 100%)		
	How often do you make mistakes in your daily work?<br>• Never<br>• Rarely<br>• Occasionally<br>• Often<br>• Very often	Single choice
	In which activities do you think mistakes occur most frequently?<br>• Taking a medical history<br>• Handling / restraining animals<br>• Handing over patients to colleagues<br>• Taking over patients from colleagues<br>• Billing / invoicing<br>• Medication administration<br>• Medication dosage<br>• Assisting in surgery<br>• Anaesthesia<br>• Interaction with animal owners (e.g. dispensing medication)<br>• Team interaction	Ranking scale
	Which of the following factors do you think most frequently contribute to errors in your work?<br>• Inadequate equipment<br>• Insufficient training / continuing professional development	Multiple choice

	• Lack of experience<br>• Lack of supervision<br>• Unclear work instructions<br>• High workload<br>• Time pressure<br>• Technical problems / failures<br>• Rushed working conditions<br>• Fatigue / exhaustion<br>• Team communication<br>• Communication with animal owners<br>• Other	
	To what extent does workload play a role in the occurrence of errors?<br>• Very large role<br>• Large role<br>• Moderate role<br>• Small role<br>• No role	Single choice
	If an error occurs, how do you deal with it?<br>• You address it openly<br>• You do not address it openly<br>• You usually try to resolve it yourself<br>• You reflect on it afterwards<br>• You have concealed errors in the past<br>• Other	Single choice
	If an error occurs, how do you and your team deal with it?<br>• Errors are openly discussed and analysed<br>• Errors are covered up<br>• Errors lead to discussions with a supervisor<br>• There is a defined protocol for dealing with errors<br>• Other	Multiple choice
	Is there a structured error reporting and management system in your organisation?<br>• Yes<br>• No<br>• You do not know	Single choice
	Do you believe that the working environment (e.g. physical space, technical equipment) contributes to errors?<br>• Yes, strong<br>• Yes, partly<br>• Neutral<br>• Small<br>• Not at all	Single choice
	How often do communication problems within the team lead to errors?<br>• Very often<br>• Often<br>• Sometimes<br>• Rarely<br>• Never	Single choice
	Do you feel safe addressing errors within your team?<br>• Always	Single choice

	• Mostly<br>• Sometimes<br>• Rarely<br>• Never	
	How well do you feel your training / continuing professional development has prepared you to prevent errors?<br>• Very well prepared<br>• Well prepared<br>• Moderately prepared<br>• Poorly prepared<br>• Very poorly prepared	Single choice
	What do you think would be good suggestions for preventing errors?	Free-text response
Working Enviroment (N=205, 100%)		
	How would you describe the current working atmosphere in your organisation?<br>• Very positive<br>• Rather positive<br>• Neutral<br>• Rather negative<br>• Very negative	Single choice
	How burdened do you feel by your work?<br>• Not burden at all<br>• Low burden<br>• Moderate burden<br>• High burden<br>• Very high burden	Single choice
	How satisfied are you with your current professional situation?<br>• Very satisfied<br>• Satisfied<br>• Neutral<br>• Dissatisfied<br>• Very dissatisfied	Single choice
	In your opinion, what could help improve the working atmosphere?	Free-text response
Demographics (N=205, 100%)		
	What is your gender identity?<br>• Female<br>• Male<br>• Diverse / non-binary<br>• No response	Single choice
	Please indicate your age (e.g. 20)	Dropdown selection

All survey questions were translated into English. Some German terms, however, are not directly transferable.
